# Characteristics of familial type 1 diabetes: effects of the relationship to the affected family member on phenotype and genotype at diagnosis

**DOI:** 10.1007/s00125-019-4952-8

**Published:** 2019-07-25

**Authors:** Maaret Turtinen, Taina Härkönen, Anna Parkkola, Jorma Ilonen, Mikael Knip, the Finnish Pediatric Diabetes Register

**Affiliations:** 1 Children’s Hospital, University of Helsinki, P.O. Box 22, (Stenbäckinkatu 11), FI-00014 Helsinki, Finland; 2 Research Program for Clinical and Molecular Metabolism, Faculty of Medicine, University of Helsinki, Helsinki, Finland; 3 Immunogenetics Laboratory, Institute of Biomedicine, University of Turku, Turku, Finland; 4 Clinical Microbiology, Turku University Hospital, Turku, Finland; 5 Folkhälsan Research Center, Helsinki, Finland; 6 Tampere Center for Child Health Research, Tampere University Hospital, Tampere, Finland

**Keywords:** Autoantibodies, Clinical characteristics, Family, HLA class II, Type 1 diabetes

## Abstract

**Aims/hypothesis:**

In previous studies, the risk of developing familial type 1 diabetes has been reported to be more than two times higher in the offspring of affected fathers than in those of affected mothers. We tested the hypothesis that index children with an affected father may have a more aggressive disease process at diagnosis than those with other affected first-degree relatives.

**Methods:**

A cross-sectional, observational study was performed using the Finnish Pediatric Diabetes Register. Clinical and metabolic characteristics, beta cell autoantibodies and HLA class II genetics were analysed from index children in Finland diagnosed before the age of 15 years between January 2003 and December 2016. Information on the presence of type 1 diabetes in first-degree relatives was collected at diagnosis using a structured questionnaire.

**Results:**

Out of 4993 newly diagnosed index children, 519 (10.4%) had familial type 1 diabetes. More than 5% (*n* = 253, 5.1%) had an affected father, 2.8% (*n* = 141) had an affected mother, 1.9% (*n* = 95) had an affected sibling and 0.6% (*n* = 30) had two or more affected family members. All clinical and metabolic variables were markedly poorer in children with sporadic vs familial diabetes. The index children with an affected father or mother were younger than those with an affected sibling (median age 7.59 vs 6.74 vs 10.73 years, respectively; *p <* 0.001). After age- and sex-adjusted analyses, index children with an affected father presented more often with ketoacidosis (9.7% vs 3.6%; *p =* 0.033) and had greater weight loss before diagnosis (3.2% vs 0%; *p =* 0.006) than those with an affected mother. Children with familial disease tested negative for all autoantibodies more often (3.5% vs 2.1%; *p =* 0.041) and had insulin autoantibodies more frequently (49.8% vs 42.2%; *p* = 0.004) than those with sporadic disease. Both major HLA risk haplotypes (DR3-DQ2 and DR4-DQ8) were more often lacking among children with sporadic vs familial disease (15.9% vs 11.2%; *p* = 0.006). The DR4-DQ8 haplotype was more frequent in the familial vs the sporadic group (75.7% vs 68.5%; *p* = 0.001) and especially among children with an affected father when compared with children with sporadic disease (77.5% vs 68.5%; *p* < 0.05). When comparing index children with affected parents diagnosed before or after the birth of the index child, a clear male preponderance was seen among the affected parents diagnosed before the birth of the index child (fathers 66.2% vs mothers 33.8%; *p* = 0.006), whereas the proportion of fathers and mothers was similar if type 1 diabetes was diagnosed after the birth of the index child.

**Conclusions/interpretation:**

The more severe metabolic derangement at diagnosis in children with sporadic type 1 diabetes compared with those with familial type 1 diabetes was confirmed. The higher frequency of diabetic ketoacidosis and increased weight loss at diagnosis in index children with an affected father compared with an affected mother support the hypothesis that paternal type 1 diabetes is associated with more severe disease in the offspring than maternal diabetes. The sex difference seen between affected parents diagnosed before and after the birth of the index child supports the hypothesis that maternal insulin treatment protects against type 1 diabetes.

**Electronic supplementary material:**

The online version of this article (10.1007/s00125-019-4952-8) contains peer-reviewed but unedited supplementary material, which is available to authorised users.

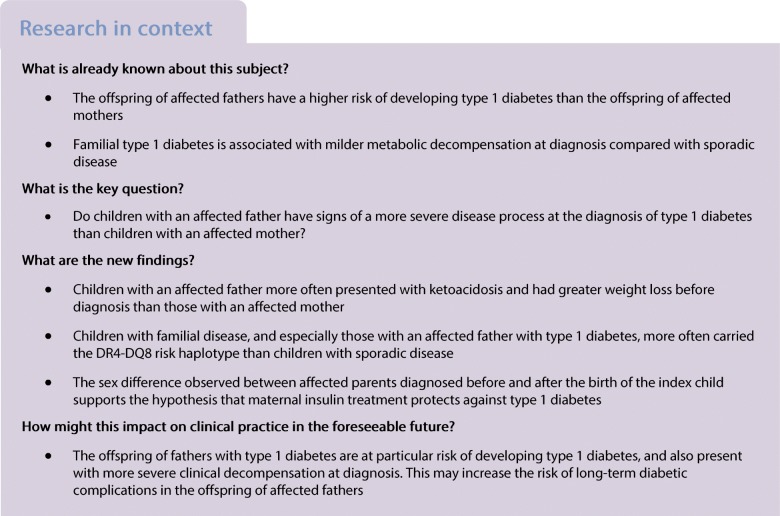



## Introduction

Warram et al first reported a higher risk of type 1 diabetes in the children of affected fathers compared with the children of affected mothers [1], and this has been confirmed in many other studies [2–9]. However, there are few recent studies, and methodological differences in study designs make the comparison of existing studies challenging. Moreover, studies have focused on the prevalence and incidence rate of familial type 1 diabetes, as well as on the risk of disease transmission from parents to offspring or comparisons between parent–offspring and sib-pair groups. A more severe metabolic derangement at diagnosis has been observed in individuals with sporadic compared with familial type 1 diabetes [4, 8, 10–13]. However, little is known about possible differences in disease presentation at diagnosis of type 1 diabetes according to who in the family is also affected by type 1 diabetes.

Despite the confirmed fact of a stronger disease transmission to offspring from an affected father compared with an affected mother, the mechanism behind this phenomenon is still poorly understood. Both genetic and environmental hypotheses have been proposed. These include preferential transmission of HLA susceptible genes from the father to the offspring [14, 15]; genomic imprinting resulting in diabetes-susceptible gene inactivation in the offspring of affected mothers but not in those of affected fathers [16]; a tolerogenic environment in insulin-treated mothers during pregnancy [1, 17]; and the selective loss of fetuses of affected mothers carrying susceptibility to type 1 diabetes by spontaneous abortion [1]. This last hypothesis of higher perinatal mortality rate in fetuses of affected mothers does not, however, appear to explain this phenomenon. Warram et al in their later work [18] found that the risk of type 1 diabetes in the offspring remained basically the same over several decades, despite a significant decrease in perinatal mortality rate over the same time period.

The aim of our study was to analyse the impact of the relationship of the family member affected by type 1 diabetes on clinical, humoral and genetic characteristics in the index child at diagnosis of type 1 diabetes. Since the risk of type 1 diabetes is two- to threefold higher among those with an affected father compared with those with an affected mother, we expected the index children with an affected father to have signs of a more aggressive disease process than those with an affected mother or sibling.

## Methods

### Study design and subjects

The Finnish Pediatric Diabetes Register (FPDR) and Sample Repository has provided clinical and biochemical information on children newly diagnosed with diabetes and their families since 2002, and covers more than 90% of those diagnosed with diabetes in Finland [19]. The coverage of the Sample Repository is approximately 70% of participants. The newly diagnosed children have been treated in 23 centres, of which five are university hospitals.

**Fig. 1:**
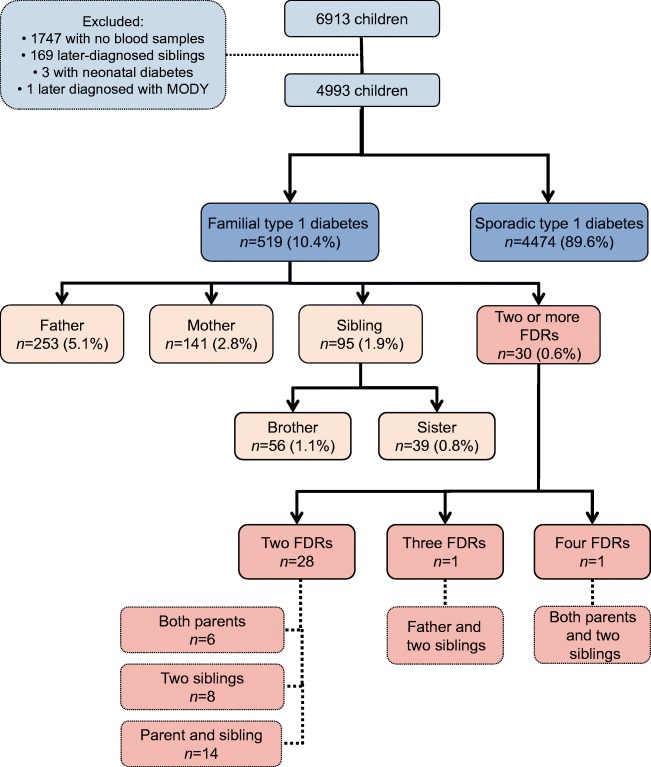
Flow diagram showing inclusion and exclusion criteria and grouping of the index children according to the relationship to the affected FDR. All children and adolescents were diagnosed between 2003 and 2016, with age at diagnosis ≤15 years

Between January 2003 and December 2016, 6913 children and adolescents diagnosed with type 1 diabetes under the age of 15 years were registered in the FPDR. We excluded 1747 children because of a lack of blood samples available for the analyses of diabetes-associated autoantibodies and HLA genotyping (Fig. 1). A comparison between the included and excluded participants has been presented in our previous work [20]. Only the first diagnosed child per family who fulfilled the inclusion criteria was included as an index child. Three children potentially suffering from neonatal diabetes (age at diagnosis <6 months) and one child later diagnosed with maturity-onset diabetes of the young were excluded. Consequently, the study cohort included 4993 children, of whom 56.6% were boys and the mean age was 8.03 years (SD 3.89, range 0.52–14.99 years).

Information on the family history of all types of diabetes was collected from the families using a structured questionnaire. First-degree relatives (FDRs) included the father, the mother and the full siblings of the affected child. The register does not provide follow-up data from families after the diagnosis. In the current analyses, we took into account the family history of type 1 diabetes in FDRs at the time of diagnosis of the index child. The study cohort was categorised into four familial subgroups based on which family member or members were affected by type 1 diabetes (father, mother, sibling or more than one affected FDR) and those with sporadic disease. Children with no information on the family history of type 1 diabetes (*n* = 57) were counted as having sporadic disease.

Written informed consent was required from a parent or a legal caretaker and from participants aged 18 years or older. Children aged 10–17 years gave informed assent. The study protocol was approved by the Ethics Committee of the Hospital District of Helsinki and Uusimaa.

We first compared children with familial and sporadic disease at the time of clinical diagnosis. A more detailed comparison between the four familial subgroups and those with sporadic disease was further performed, and in case of significant differences, paired comparisons by groups were also performed.

### Markers of metabolic decompensation at diagnosis

Local laboratories analysed blood pH and the levels of plasma glucose and β-hydroxybutyrate at diagnosis. Standardised HbA_1c_ values were available only from those diagnosed after 2012. The clinician in charge of the initial treatment of the index child assessed weight loss and level of consciousness at the time of hospital admission. The duration of classic symptoms before diagnosis was obtained by interviewing the parents using a structured questionnaire. Ketoacidosis was defined as blood pH <7.30 and severe ketoacidosis as blood pH <7.10 at diagnosis.

### Autoantibody assays and HLA genotyping

The biochemical autoantibodies: insulin autoantibodies (IAA), antibodies to GAD (GADA), islet antigen 2 (IA-2A) and zinc transporter 8 (ZnT8A) were analysed with specific radiobinding assays described previously [21, 22]. Levels of islet cell antibodies (ICA) were quantified with indirect immunofluorescence of pancreatic tissue obtained from a blood group O (0) donor [23]. Since exogenous insulin treatment may induce insulin antibodies, we excluded those with serum samples taken later than 30 days after diagnosis (255 of 4993, 5.1%) for the autoantibody analyses. We have previously observed that insulin antibody levels 1 month after the diagnosis correlate more strongly with the IAA titers at diagnosis than with insulin antibody levels 3 months later. Typing of the major HLA DR-DQ haplotypes was performed with PCR-based lanthanide-labelled hybridisation and time-resolved fluorometry detection [24]. Detailed information on the assays is in the electronic supplementary material (ESM) Methods.

### Statistical analysis

Statistical analyses were performed using SPSS software (SPSS Statistics 24; IBM, Chicago, IL, USA) and the R 3.4.0 package (https://cran.r-project.org/). Frequencies in each study group were compared using cross-tabulation and the χ^2^ or Fisher’s exact tests. We used Pearson’s χ^2^ test for cross-tabulating polytomous variables with continuity correction when appropriate. Differences in levels of normally distributed variables were analysed using Student’s *t* test. Non-parametric variables were tested using the Mann–Whitney *U* test and Kruskal–Wallis test. Adjusted analyses for confounding factors were performed using logistic/ordinal/multinomial regression for dichotomous/ordinal/categorical variables and quantile regression in R (package quantreg) for skewed variables. A *p* value of 0.05 or less was considered statistically significant. Bonferroni’s correction for multiple comparisons was not applied due to its overly conservative nature. Multiplicity issues were taken into account in cautious interpretation of the results.

## Results

### Demographic information

At diagnosis, 519/4993 (10.4%) children had at least one FDR with type 1 diabetes. The proportion of index children with an affected father was 1.8 and 2.7 times higher compared with those with an affected mother (*p <* 0.001) or sibling (*p* < 0.001) (*n* = 253 [5.1%] vs *n* = 141 [2.8%] and *n* = 95 [1.9%], respectively). A higher proportion of index children had an affected mother than an affected sibling (*p* < 0.003). Only 30 index children (0.6%) had two or more affected FDRs with type 1 diabetes (Fig. 1). The number of children in each family and the birth order of the index child in relation to the presence of an affected father or mother are presented in the ESM Results section and ESM Table 1.

**Table 1:** Demographic, metabolic, immunological and genetic markers in children with familial and sporadic type 1 diabetes

Variable	*n*	Familial, *n*=519 (10.4%)	Sporadic, *n*=4474 (89.6%)	*p* value	Adjusted *p* value^a^
Demographics					
Age at diagnosis, years, mean ± SD	4993	7.84 ± 3.88	8.05 ± 3.89	0.229	
Sex, male, % (95% CI)	4993	55.7 (51.4, 60.0)	56.7 (55.2, 58.1)	0.705	
Pubertal, % (95% CI)	3764	16.9 (13.2, 20.6)	17.2 (15.9, 18.4)	0.941	0.445
Metabolic decompensation at diagnosis					
Duration of symptoms, %	4614			<0.001	<0.001
No symptoms		2.1	0.8		
< 1 week		38.1	20.8		
1–4 weeks		46.6	58.7		
> 4 weeks		13.1	19.7		
Impaired consciousness, % (95% CI)	4784	1.6 (0.5, 2.7)	5.9 (5.2, 6.6)	<0.001	<0.001
Ketoacidosis, % (95% CI)	4817	7.8 (5.4, 10.1)	19.1 (18.0, 20.3)	<0.001	<0.001
Severe ketoacidosis, % (95% CI)	4817	2.4 (1.1, 3.7)	4.9 (4.2, 5.5)	0.017	0.018
Weight loss, %, median (range)	4610	2.0 (0–25.3)	5.6 (0–40.0)	<0.001	<0.001
pH, median (range)	4817	7.40 (6.93–7.57)	7.38 (6.72–7.54)	<0.001	<0.001
β-Hydroxybutyrate, mmol/l, median (range)	4384	0.50 (0–18.0)	1.90 (0–27.0)	<0.001	<0.001
Plasma glucose, mmol/l, median (range)	4869	20.8 (3.6–63.7)	24.2 (3.2–97.6)	<0.001	<0.001
HbA_1c_, mmol/mol, median (range)	841	76.0 (38.0–141.5)	92.0 (36.0–189.0)	<0.001	<0.001
HbA_1c_, %, median (range)	841	9.1 (5.6–15.1)	10.6 (5.4–19.4)	<0.001	<0.001
Autoantibodies					
ICA, % (95% CI)	4738	90.9 (88.3, 93.5)	91.8 (91.0, 92.7)	0.535	0.400
ICA, JDFU, median (range)	4347	64.0 (3.0–2620.0)	64.0 (3.0–5120.0)	0.894	0.585
IAA, % (95% CI)	4738	49.8 (45.3, 54.2)	42.2 (40.7, 43.7)	0.002	0.004
IAA, RU, median (range)	2037	10.5 (2.9–484.9)	10.2 (2.8–7809.0)	0.771	0.329
IA-2A, % (95% CI)	4738	75.4 (71.6, 79.2)	75.0 (73.7, 76.3)	0.890	0.806
IA-2A, RU, median (range)	3556	104.2 (0.8–223.2)	105.8 (0.8–553.3)	0.768	0.811
GADA, % (95% CI)	4738	67.1 (63.0, 71.3)	66.3 (64.8, 67.7)	0.735	0.679
GADA, RU, median (range)	3144	43.3 (5.4–3800.0)	35.7 (5.4–24,849.0)	0.245	0.080
ZnT8A, % (95% CI)	4738	66.3 (62.1, 70.5)	69.8 (68.4, 71.1)	0.132	0.155
ZnT8A, RU, median (range)	3289	12.0 (0.5–186.7)	12.1 (0.5–1201.9)	0.753	0.767
Number of positive antibodies, median (mean)	4738	4 (3.50)	4 (3.45)	0.252	0.183
Number of positive biochemical antibodies, median (mean)	4738	3 (2.59)	3 (2.53)	0.182	0.211
Autoantibody negative, % (95% CI)	4738	3.5 (1.9, 5.2)	2.1 (1.7, 2.6)	0.079	0.041
Positivity for multiple (≥2) autoantibodies, % (95% CI)	4738	92.8 (90.5, 95.1)	92.5 (91.7, 93.2)	0.875	0.911
Genetics	4993				
DR3-DQ2/DR4-DQ8, % (95% CI)		23.7 (20.0, 27.4)	21.1 (19.9, 22.2)	0.182	0.186
DR3-DQ2/x^b^, % (95% CI)		13.1 (10.2, 16.0)	15.6 (14.5, 16.6)	0.156	0.143
DR4-DQ8/y^c^, % (95% CI)		52.0 (47.7, 56.3)	47.5 (46.0, 48.9)	0.055	0.049
x^b^/y^c^, % (95% CI)		11.2 (8.5, 13.9)	15.9 (14.8, 17.0)	0.006	0.006
DR3-DQ2, % (95% CI)		36.8 (32.7, 41.0)	36.6 (35.2, 38.0)	0.979	0.975
DR4-DQ8, % (95% CI)		75.7 (72.0, 79.4)	68.5 (67.1, 69.9)	0.001	0.001
Risk group, %				0.157	0.162
0		0.4	0.8		
1		1.7	2.1		
2		13.5	16.2		
3		20.4	23.0		
4		40.3	36.8		
5		23.7	21.1		

Categorical variables are presented as % (95% CI) and continuous variables as mean (SD) or median (range)

For comparing frequencies, cross tabulation and χ^2^ statistics with continuity correction or Fisher’s exact test when appropriate were used. Differences in levels of parametric variables were analysed with Student’s *t* test, and Kruskal–Wallis test or Mann–Whitney *U* test for non-parametric variables

Adjustment for confounding factors was performed using logistic/ordinal/multinomial regression for dichotomous/ordinal/categorical variables and quantile regression in R for skewed variables

^a^Adjusted for sex and age at diagnosis

^b^x ≠ DR4-DQ8

^c^y ≠ DR3-DQ2

JDFU, Juvenile Diabetes Foundation units; RU, relative units

**Fig. 2:**
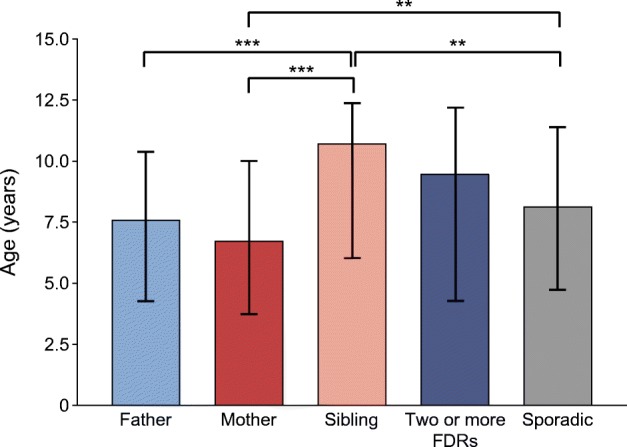
Median age (interquartile range) at diagnosis of type 1 diabetes in children with different family members with diabetes and children with sporadic type 1 diabetes. **p<0.01, ***p<0.001 (adjusted for sex and age at diagnosis). Significance was evaluated using quantile regression in R

Similar age at diagnosis and a similar male:female ratio was found between children with familial and sporadic disease (Table 1). A younger age at diagnosis was seen in index children with an affected father or mother compared with those with an affected sibling (median 7.59 and 6.74 years vs 10.73 years, respectively; *p <* 0.001, Fig. 2). There was a notable male preponderance (76.7%) among those with two or more affected FDRs compared with the male:female ratio in the other groups (*p* = 0.061, Table 2). In addition, the proportion of boys among the offspring of affected mothers (48.2%) diverged from that in the whole study population (56.6%), while the proportion of boys was similar (56–57%) in the other familial subgroups. Both sexes had a father with type 1 diabetes (5.1% of boys vs 5.0% of girls; *p* = 0.80) and a sibling with type 1 diabetes (1.9% for both sexes; *p* = 0.88) equally often. However, the proportion of girls with an affected mother was significantly higher than that of boys (3.4% vs 2.4%; *p* = 0.04) (see ESM Results). The proportion of children with two or more affected FDRs was 0.8% among boys and 0.3% among girls (*p* = 0.03).

### Metabolic decompensation at diagnosis

**Fig. 3:**
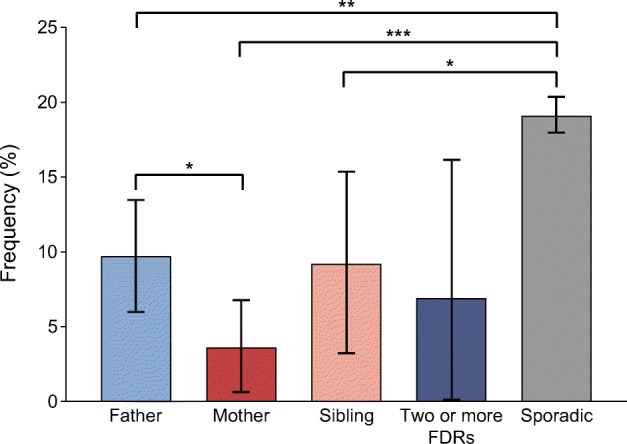
Frequency of diabetic ketoacidosis (95% CI) at diagnosis of type 1 diabetes in children with different family members with diabetes and children with sporadic type 1 diabetes. *p<0.05, **p<0.01, ***p<0.001 (adjusted for sex and age at diagnosis). Significance was evaluated using logistic regression analysis

After age- and sex-adjusted analyses, children with sporadic disease had poorer values for all clinical and metabolic variables than children with familial disease (Table 1). Index children with an affected father had a longer duration of classic symptoms compared with other familial subgroups (Table 2). They also presented more often with ketoacidosis (9.7% vs 3.6%; *p =* 0.033, Fig. 3) and had greater weight loss before diagnosis, compared with those with an affected mother (Table 2). The measured laboratory values at diagnosis were similar in the different familial subgroups.

**Table 2:** Demographic, clinical and metabolic characteristics in the four familial subgroups and in the sporadic group at diagnosis of type 1 diabetes

Variable	*n*	1. Affected father, *n*=253 (5.1%)	2. Affected mother, *n*=141 (2.8%)	3. Affected sibling, *n*=95 (1.9%)	4. ≥2 affected family members, *n*=30 (0.6%)	5. Sporadic, *n*=4474 (89.6%)	*p* value	Adjusted *p* value^a^
Demographics								
Sex, male, % (95% CI)	4993	57.3 (51.2, 63.4)	48.2 (40.0, 56.5)	55.8 (45.8, 65.8)	76.7 (61.5, 91.8)	56.7 (55.2, 58.1)	0.061	
Pubertal, % (95% CI)	3764	15.4 (10.3, 20.4)	9.6 (4.2, 15.1)	30.4 (19.6, 41.3)	26.3 (6.5, 46.1)	17.2 (15.9, 18.4)	0.005	0.551
Metabolic decompensation at diagnosis								
Duration of symptoms, %	4614						<0.001	<0.001
No symptoms		1.3	1.5	4.7	3.7	0.8		
<1 week		31.0	44.6	41.9	55.6	20.8		
1–4 weeks		52.4	42.3	39.5	40.7	58.7		
>4 weeks		15.3	11.5	14.0	0	19.7		
								1 vs 3: 0.015
								1 vs 4: 0.007
								1 vs 5: 0.006
								2 vs 4: 0.049
								2 vs 5: <0.001
								3 vs 5: <0.001
								4 vs 5: <0.001
Impaired consciousness, % (95% CI)	4784	1.6 (0, 3.2)	0.7 (0, 2.2)	2.2 (0, 5.2)	3.6 (0, 10.4)	5.9 (5.2, 6.6)	0.003	0.010
								1 vs 5: 0.010
								2 vs 5: 0.036
Weight loss, %, median (range)	4610	3.2 (0–21.3)	0 (0–19.7)	1.6 (0–25.3)	3.4 (0–14.8)	5.6 (0–40.0)	<0.001	<0.001
								1 vs 2: 0.006
								1 vs 5: <0.001
								2 vs 4: 0.034
								2 vs 5: <0.001
								3 vs 4: 0.045
								3 vs 5: <0.001
pH, median (range)	4817	7.40 (6.97–7.57)	7.40 (7.00–7.53)	7.40 (6.93–7.46)	7.40 (7.09–7.44)	7.38 (6.72–7.54)	<0.001	<0.001
								1 vs 5: <0.001
								2 vs 5: <0.001
								3 vs 5: <0.001
								4 vs 5: 0.007
β-Hydroxybutyrate, mmol/l, median (range)	4384	0.69 (0–12.1)	0.30 (0–9.5)	0.50 (0–18.0)	0.70 (0–17.4)	1.90 (0–27.0)	<0.001	<0.001
								1 vs 5: <0.001
								2 vs 5: <0.001
								3 vs 5: <0.001
								4 vs 5: <0.001
Plasma glucose, mmol/l, median (range)	4869	21.5 (3.9–62.8)	19.7 (3.6–47.8)	20.0 (4.6–56.5)	20.8 (9.6–63.7)	24.2 (3.2–97.6)	<0.001	<0.001
								1 vs 5: <0.001
								2 vs 5: <0.001
								3 vs 5: <0.001
HbA_1c_, mmol/mol, median (range)	841	75.5 (38.0–141.5)	78.0 (40.0–132.8)	82.0 (39.9–121.0)	67.0 (64.0–96.0)	92.0 (36.0–189.0)	<0.001	<0.001
								1 vs 5: <0.001
								2 vs 4: 0.025
								2 vs 5: 0.016
HbA_1c_, %, median (range)	841	9.1 (5.6–15.1)	9.3 (5.8–14.3)	9.7 (5.8–13.2)	8.3 (8.0–10.9)	10.6 (5.4–19.4)	<0.001	0.002
								1 vs 5: <0.01
								2 vs 4: 0.022
								2 vs 5: 0.013

Categorical variables are presented as % (95% CI) and continuous variables as median (range)

In case of significant differences in the analyses between the four familial subgroups and those with sporadic disease, paired comparisons by groups were also performed. Only significant *p* values are presented from the paired analyses

For comparing frequencies in each study group, cross tabulation and χ^2^ statistics with continuity correction or Fisher’s exact test when appropriate were used. Differences in levels were determined using Kruskal–Wallis test or the Mann–Whitney *U* test

Adjustment for confounding factors was performed using logistic/ordinal/multinomial regression for dichotomous/ordinal/categorical variables and quantile regression in R for skewed variables

^a^Adjusted for sex and age at diagnosis

### Autoantibodies

When adjusted for age and sex, autoantibody negativity was more frequent among children with familial vs sporadic disease (Table 1), and a similar difference was seen especially among those with more than one affected FDR compared with those with sporadic disease, although it did not reach statistical significance after adjustment (*p* = 0.091) (Table 3). The frequency of IAA was higher in the familial group, but all other autoantibody frequencies and titres were similar in familial compared with sporadic groups, and no differences were detected in the number of positive autoantibodies (Table 1).

**Table 3:** Autoantibody positivity, levels of various autoantibodies in positive samples and number of positive autoantibodies in familial groups and in the sporadic group at diagnosis of type 1 diabetes

Autoantibodies	*n*	1. Affected father, *n*=253 (5.1%)	2. Affected mother, *n*=141 (2.8%)	3. Affected sibling, *n*=95 (1.9%)	4. ≥2 affected family members, *n*=30 (0.6%)	5. Sporadic, *n*=4474 (89.6%)	*p* value	Adjusted *p* value^a^
ICA, % (95% CI)	4738	89.5 (85.5, 93.4)	93.3 (89.1, 97.5)	92.8 (87.2, 98.3)	86.2 (73.3, 98.8)	91.8 (91.0, 92.3)	0.487	0.451
ICA, JDFU, median (range)	4347	64.0 (3.0–2620.0)	49.0 (3.0–2048.0)	64.0 (4.0–1024.0)	47.0 (5.0–1024.0)	64.0 (3.0–5120.0)	0.530	0.866
IAA, % (95% CI)	4738	49.8 (43.4, 56.2)	47.4 (39.0, 55.8)	51.8 (41.1, 62.6)	55.2 (37.1, 73.3)	42.2 (40.7, 43.7)	0.027	0.003
								2 vs 3: 0.018
								3 vs 5: <0.01
IAA, RU, median (range)	2037	9.9 (2.9–260.0)	13.5 (3.2–338.7)	9.0 (2.9–48.8)	10.7 (3.5–484.9)	10.2 (2.8–7809.0)	0.437	0.447
IA-2A, % (95% CI)	4738	74.3 (68.7, 79.8)	80.0 (73.3, 86.7)	74.7 (65.3, 84.1)	65.5 (48.2, 82.8)	75.0 (73.7, 76.3)	0.515	0.433
IA-2A, RU, median (range)	3556	102.1 (0.9–223.2)	99.7 (1.1–202.5)	110.3 (0.8–185.0)	121.2 (18.3–186.6)	105.8 (0.8–553.3)	0.067	0.032
								4 vs 5: 0.058
GADA, % (95% CI)	4738	67.1 (61.1, 73.1)	67.4 (59.5, 75.3)	69.9 (60.0, 79.7)	58.6 (40.7, 76.5)	66.3 (64.8, 67.7)	0.848	0.942
GADA, RU, median (range)	3144	49.8 (5.4–3800.0)	43.3 (6.3–277.4)	37.2 (6.4–349.4)	35.6 (7.4–185.3)	35.7 (5.4–24,849.0)	0.826	0.192
ZnT8A, % (95% CI)	4738	65.0 (58.9, 71.1)	63.7 (55.6, 71.8)	75.9 (66.7, 85.1)	62.1 (44.4, 79.7)	69.8 (68.4, 71.1)	0.141	0.243
ZnT8A, RU, median (range)	3289	11.1 (0.5–186.7)	10.9 (0.6–138.4)	16.1 (0.6–126.8)	21.9 (0.6–93.0)	12.1 (0.5–1201.9)	0.428	0.985
Number of positive antibodies, median (mean)	4738	4 (3.46)	4 (3.52)	4 (3.65)	4 (3.28)	4 (3.45)	0.835	0.436
Number of positive biochemical antibodies, median (mean)	4738	3 (2.56)	3 (2.59)	3 (2.72)	3 (2.41)	3 (2.53)	0.831	0.483
Autoantibody negative, % (95% CI)	4738	3.0 (0.8, 5.1)	3.0 (0.1, 5.8)	3.6 (0, 7.6)	10.3 (0, 21.4)	2.1 (1.7, 2.6)	0.049	0.091
Positivity for multiple (≥2) autoantibodies, % (95% CI)	4738	92.0 (88.5, 95.4)	94.1 (90.1, 98.1)	95.2 (90.6, 99.8)	86.2 (73.7, 98.8)	92.5 (91.7, 93.2)	0.542	0.552

Categorical variables are presented as % (95% CI) and continuous variables as median (range)

In case of significant differences in the analyses between the four familial subgroups and those with sporadic disease, paired comparisons by groups were also performed. Only significant *p* values are presented from the paired analyses

For comparing frequencies in each study group, cross tabulation and χ^2^ statistics with continuity correction or Fisher’s exact test when appropriate were used. Differences in levels were determined using Kruskal–Wallis test or the Mann–Whitney *U* test

Adjustment for confounding factors was performed using logistic/ordinal/multinomial regression for dichotomous/ordinal/categorical variables and quantile regression in R for skewed variables

^a^Adjusted for sex and age at diagnosis

JDFU, Juvenile Diabetes Foundation unit; RU, relative units

### HLA genetics

After adjusting for age and sex, the risk haplotype DR4-DQ8 was more common in the familial vs sporadic group (Table 1), and a higher prevalence of DR4-DQ8 heterozygosity as well as DR4-DQ8 homozygosity was observed in those with an affected father vs those with sporadic disease, in particular (Table 4). The genotypes missing both major risk haplotypes (DR3-DQ2 and DR4-DQ8) were more common in the sporadic group compared with the familial group (Table 1). There was no difference in the proportions of genetic risk groups between children with familial or sporadic disease, or between different familial subgroups (Tables 1 and 4).

**Table 4:** Frequencies of HLA risk genotypes and haplotypes at diagnosis in familial groups and children with sporadic diabetes (*n* = 4993)

Genetics, % (95% CI)	1. Affected father, *n*=253 (5.1%)	2. Affected mother, *n*=141 (2.8%)	3. Affected sibling, *n*=95 (1.9%)	4. ≥2 affected family members, *n*=30 (0.6%)	5. Sporadic, *n*=4474 (89.6%)	*p* value	Adjusted *p* value^a^
DR3-DQ2/DR4-DQ8	21.3 (16.3, 26.4)	23.4 (16.4, 30.4)	29.5 (20.3, 38.6)	26.7 (10.8, 42.5)	21.1 (19.9, 22.2)	0.306	0.238
DR3-DQ2/x^b^	11.1 (7.2, 14.9)	17.0 (10.8, 23.2)	12.6 (6.0, 19.3)	13.3 (1.2, 25.5)	15.6 (14.5, 16.6)	0.320	0.313
DR4-DQ8/y^c^	56.1 (50.0, 62.2)	51.1 (42.8, 59.3)	42.1 (32.2, 52.0)	53.3 (35.5, 71.2)	47.5 (46.0, 48.9)	0.052	0.048
							1 vs 3: <0.05
							1 vs 5: <0.05
x^b^/y^c^	11.5 (7.5, 15.4)	8.5 (3.9, 13.1)	15.8 (8.5, 23.1)	6.7 (0, 15.6)	15.9 (14.8, 17.0)	0.030	0.044
							2 vs 5: <0.05
DR3-DQ2 homozygosity	3.2 (1.0, 5.3)	2.8 (0.1, 5.6)	3.2 (0, 6.7)	3.3 (0, 9.8)	3.0 (2.5, 3.5)	0.971	1.000
DR4-DQ8 homozygosity	15.0 (10.6, 19.4)	10.6 (5.5, 15.7)	8.4 (2.8, 14.0)	10.0 (0, 20.7)	7.6 (6.9, 8.4)	0.001	0.001
							1 vs 5: 0.001
DR3-DQ2	32.4 (26.6, 38.2)	40.4 (32.3, 48.5)	42.1 (32.2, 52.0)	40.0 (22.5, 57.5)	36.6 (35.2, 38.0)	0.381	0.336
DR4-DQ8	77.5 (72.3, 82.6)	74.5 (67.3, 81.7)	71.6 (62.5, 80.6)	80.0 (65.7, 94.3)	68.5 (67.1, 70.0)	0.012	0.015
							1 vs 5: <0.05

Variables are presented as % (95% CI)

In case of significant differences in the analyses between the four familial subgroups and those with sporadic disease, paired comparisons by groups were also performed. Only significant *p* values are presented from the paired analyses

For comparing frequencies in each study group, cross tabulation and χ^2^ statistics with continuity correction or Fisher’s exact test when appropriate were used. Differences in levels were determined using Kruskal–Wallis test or the Mann–Whitney *U* test

Adjustment for confounding factors was performed using logistic/ordinal/multinomial regression for dichotomous/ordinal/categorical variables

^a^Adjusted for sex and age at diagnosis

^b^x ≠ DR4-DQ8

^c^y ≠ DR3-DQ2

### Timing of diagnosis in the affected parent in relation to disease characteristics in the index child

We compared offspring with parents diagnosed with type 1 diabetes before and after the birth of the index child (Table 5). In these analyses, we included all the index children known to have affected parents, and also those classified into the group of two or more affected FDRs in previous analyses. If both parents of an index child had type 1 diabetes (six children) then we took into account only the information from the affected mothers. Thus, in 349 cases (87%) the parent was diagnosed before the birth of the index child, and in 52 cases (13%) the parent was diagnosed after the birth of the index child.

**Table 5:** Demographic, metabolic, immunological and genetic markers in children with parents diagnosed with type 1 diabetes before and after the birth of the index child

Variable	*n*	Before, *n*=349 (87.0%)	After, *n*=52 (13.0%)	*p* value	Adjusted *p* value^a^
Demographics					
Age at diagnosis, years, median (range)	401	6.98 (0.59–14.96)	9.57 (1.60–14.94)	0.001	
Sex, male, % (95% CI)	401	54.4 (49.2, 59.7)	65.4 (52.5, 78.3)	0.183	
Affected parent, father, % (95% CI)	401	66.2 (61.2, 71.2)	50.0 (36.4, 63.6)	0.034	0.006
Pubertal, % (95% CI)	313	12.1 (8.3, 15.9)	31.3 (15.2, 47.3)	0.007	0.964
Metabolic decompensation at diagnosis					
Duration of symptoms, %	368			0.962	0.807
No symptoms		1.3	0		
<1 week		37.3	36.7		
1–4 weeks		47.6	51.0		
>4 weeks		13.8	12.2		
Impaired consciousness, % (95% CI)	386	1.8 (0.4, 3.2)	0	1.000	0.998
Ketoacidosis, % (95% CI)	393	7.9 (5.1, 10.8)	5.8 (0, 12.1)	0.782	0.645
Weight loss, kg, median (range)	368	0.30 (0–15.0)	1.10 (0–7.0)	0.065	0.442
Weight loss, %, median (range)	367	1.6 (0–21.3)	3.8 (0–19.7)	0.195	0.316
pH, median (range)	393	7.40 (6.97–7.54)	7.40 (7.20–7.45)	0.303	0.269
β-Hydroxybutyrate, mmol/l, median (range)	346	0.50 (0–12.1)	0.49 (0–17.4)	0.960	0.850
Plasma glucose, mmol/l, median (range)	396	21.7 (3.6–62.8)	20.3 (4.8–63.7)	0.417	0.452
HbA_1c_, mmol/mol, median (range)	63	72.0 (38.0–141.5)	107.0 (71.0–132.8)	0.007	0.007
HbA_1c_, %, median (range)	63	8.7 (5.6–15.1)	12.0 (8.7–14.3)	0.006	0.007
Autoantibodies					
ICA, % (95% CI)	379	90.3 (87.1, 93.5)	92.0 (84.5, 99.5)	1.000	0.380
ICA, JDFU, median (range)	343	49.0 (3.0–2620.0)	64.0 (4.0–2048.0)	0.463	0.963
IAA, % (95% CI)	379	52.0 (46.6, 57.4)	32.0 (19.1, 44.9)	0.013	0.163
IAA, RU, median (range)	187	10.6 (2.9–484.9)	14.0 (3.0–338.7)	0.197	0.463
IA-2A, % (95% CI)	379	74.5 (69.8, 79.2)	88.0 (79.0, 97.0)	0.055	0.022
IA-2A, RU, median (range)	289	104.4 (0.9–202.5)	87.1 (1.4–157.5)	0.201	0.375
GADA, % (95% CI)	379	67.5 (62.4, 72.5)	62.0 (48.5, 75.5)	0.545	0.381
GADA, RU, median (range)	253	45.4 (5.4–3800.0)	47.9 (6.3–277.4)	0.782	0.385
ZnT8A, % (95% CI)	379	62.6 (57.4, 67.8)	76.0 (64.2, 87.8)	0.092	0.115
ZnT8A, RU, median (range)	244	10.4 (0.5–186.7)	17.0 (0.6–138.4)	0.396	0.297
Number of positive antibodies, median (mean)	379	4 (3.47)	4 (3.50)	0.701	0.292
Number of positive biochemical antibodies, median (mean)	379	3 (2.57)	3 (2.58)	0.810	0.932
Autoantibody negative, % (95% CI)	379	3.0 (1.2, 4.9)	6.0 (0, 12.6)	0.393	0.709
Positivity for multiple (≥2) autoantibodies, % (95% CI)	379	92.7 (89.9, 95.5)	94.0 (87.4, 1)	1.000	0.373
Genetics	401				
DR3-DQ2/DR4-DQ8, % (95% CI)		22.9 (18.5, 27.3)	19.2 (8.5, 29.9)	0.677	0.483
DR3-DQ2/x^b^, % (95% CI)		14.0 (10.4, 17.7)	9.6 (1.6, 17.6)	0.513	0.465
DR4-DQ8/y^c^, % (95% CI)		53.6 (48.3, 58.8)	51.9 (38.3, 65.5)	0.940	0.923
x^b^/y^c^, % (95% CI)		9.5 (6.4, 12.5)	19.2 (8.5, 29.9)	0.059	0.069
DR3-DQ2, % (95% CI)		37.0 (31.9, 42.0)	28.8 (16.5, 41.2)	0.325	0.261
DR4-DQ8, % (95% CI)		76.5 (72.1, 81.0)	71.2 (58.8, 83.5)	0.505	0.424
Risk group, %				0.125	0.404
0		0.6	0		
1		1.1	3.8		
2		11.5	23.1		
3		22.3	15.4		
4		41.5	38.5		
5		22.9	19.2		

Categorical variables are presented as % (95% CI) and continuous variables as median (range)

For comparing frequencies, cross tabulation and χ^2^-statistics with continuity correction or Fisher’s exact test when appropriate were used. Differences in levels were determined using Kruskal–Wallis test or the Mann–Whitney *U* test. Adjustment for confounding factors was performed using logistic/ordinal/multinomial regression for dichotomous/ordinal/categorical variables and quantile regression in R for skewed variables

^a^Adjusted for sex and age at diagnosis

^b^x ≠ DR4-DQ8

^c^y ≠ DR3-DQ2

JDFU, Juvenile Diabetes Foundation unit; RU, relative units

There were no significant differences in the sex distribution of the index children between the groups. In contrast, a clear predominance of affected men was seen among the affected parents diagnosed before the birth of the index child (66%), whereas the proportion of affected fathers and mothers was similar when their type 1 diabetes was diagnosed after the birth of the index child. Index children were younger if the parent was diagnosed before vs after the birth of the index child. A similar difference was seen in subanalyses of index children with an affected mother (median age 5.57 vs 9.77 years, *p* < 0.001), but not in subanalyses of index children with an affected father (data not shown). After adjusting for age and sex, median HbA_1c_ values at diagnosis of type 1 diabetes were higher in those children whose parents were diagnosed after the birth of the index child, whereas no other significant differences were observed in the metabolic characteristics or in the risk profile of HLA genetics between the two groups. Apart from higher IA-2A frequencies in children with affected parents diagnosed after the birth of the index child, no other differences were found between the groups in the autoantibody profile.

## Discussion

In this cross-sectional nationwide study, a positive family history of type 1 diabetes was observed in 10.4% of participating children at the time of diagnosis. This frequency is in line with previous reports, in which the proportion has varied between 9 and 12% [4, 6, 9, 12, 13, 25–27]. As expected, the number of children with an affected father was about twofold higher than that of children with an affected mother [2, 5, 7, 8, 26]. The proportion of index children with an affected sibling (1.9%) was lower than previously observed in Finnish and Danish studies (⁓5%) [4, 6, 13]. This may be due to the fact that siblings diagnosed after the index child were not considered in the current study. The proportion of those with an affected sibling was based on all index children, irrespective of the number of known siblings. Moreover, index children with two or more affected FDRs, at least one being a sibling, were categorised into the group of multiple affected family members and not included in the group of affected siblings. In the Childhood Diabetes in Finland (DiMe) study, the categorisation was similar to that used in the current study and the proportion of index children with a sibling with type 1 diabetes (2.6%) was closer to our finding [28]. We cannot disregard the potential effect of a decreasing birth rate and reduced family size seen in Finland on the number of siblings.

We observed a younger age at diagnosis in those with an affected father or mother than in those with an affected sibling. However, no difference was observed in age at diagnosis between children with familial and sporadic disease, similar to most previous studies [4, 9, 25, 29]. As found in a Swedish register-based study, index individuals with an affected sibling were older than those in other familial groups and those with sporadic disease [9]. In the current study, the transmission of type 1 diabetes from an affected mother to a daughter was more frequent than to a son, whereas almost the same frequency of boys and girls had an affected father or a sibling. In addition, a clear majority of those with two or more affected FDRs were boys. This was not, however, statistically significant because of the small number of children in this group. In contrast to our findings, it has been speculated that the disease transmission rate is higher in offspring of the opposite sex to that of the diabetic parent [2, 5]. Most previous studies have not, however, supported this hypothesis, showing no differences [1, 4, 9, 29]. The DiMe study reported that if the affected parent and the offspring shared the same sex then the risk of developing type 1 diabetes was higher in sons compared with daughters; when the parent and the offspring were of the opposite sex, the risk was higher in daughters [2]. Accordingly, disease transmission was higher from an affected father than from an affected mother. In another study, fathers were more likely to pass the disease to their daughters than to their sons, but a similar effect was not seen for mothers [5].

As previously shown, those without type 1 diabetes in the immediate family had more severe metabolic decompensation at diagnosis than those with familial disease [4, 8, 10–13]. The observation of a lack of differences in metabolic control between those with familial and sporadic disease 1 year after diagnosis [10] suggests that increased parental awareness of diabetes-related symptoms and/or the possibility of self-monitoring blood glucose without any delay in the families with a previously affected family member, rather than differences in the disease pathogenesis, explain this phenomenon. A higher frequency of ketoacidosis and increased weight loss at diagnosis was observed in index children with an affected father vs an affected mother. The results lend support to our hypothesis that paternal type 1 diabetes is associated with more severe disease in the offspring than maternal type 1 diabetes. To our knowledge, this is the first observation of an association between paternal type 1 diabetes and a poorer metabolic status in the offspring at diagnosis.

Since the FPDR does not collect data on the social status of families, it was not possible to look for associations between the familial environment and lifestyle and the disease presentation in the index child. Whether the child is living with both parents or with a single parent, often the mother, may affect the recognition of diabetes-related symptoms prior to diagnosis. It is possible that mothers are still the primary caregivers, responsible for children’s health issues and providing information to clinicians. Whether this could have an effect on the reported findings, for example the longer duration of classic symptoms seen in those with an affected father compared with the other familial subgroups, remains open.

The frequency of no detectable autoantibodies at diagnosis was higher in children with familial vs sporadic disease, and especially in those with multiple affected FDRs. This raises the possibility of monogenic diabetes in some children with affected family members. This issue is currently being analysed in the FPDR population. The IAA frequency was significantly higher in children with familial vs sporadic disease, while no differences were observed in other autoantibody titres or frequencies, or in the number of positive autoantibodies. Lebenthal et al reported similar results for IAA in their study [12]. In addition, they found that individuals with familial disease tested positive for three autoantibodies more often than those with sporadic disease. However, most studies have not found an association between a positive family history of type 1 diabetes and the presence of diabetes-related autoantibodies [4, 8, 30]. Although our results from the autoantibody analyses do not support the theory of a more aggressive, organ-specific immune response in index children with an affected father, previous studies in at-risk individuals have pointed in that direction. Verge et al reported that the offspring of a father with type 1 diabetes were more likely to seroconvert to positivity for diabetes-related autoantibodies than the offspring of an affected mother [31]. Similarly, the risk of developing multiple islet autoantibody positivity tended to be higher in the offspring of affected fathers vs affected mothers in the BABYDIAB study [32]. Moreover, in that study the risk of multiple autoantibodies, as well as the risk of developing type 1 diabetes, was strongly associated with the presence of multiple FDRs with type 1 diabetes. This is, in a way, in contrast to our finding of a higher frequency of no detectable autoantibodies described above. However, as the FPDR does not include any samples from the period before the diabetes diagnosis, we cannot exclude the possibility that children with familial disease had autoantibodies earlier in the disease process but were already antibody negative at diagnosis due to aggressive beta cell loss. In the recent Environmental Determinants of Diabetes in the Young study, the risk of progression from multiple autoantibodies to type 1 diabetes was not related to the presence of an FDR with type 1 diabetes compared with the general population series after 8 years of follow-up, but the progression rate was more rapid in individuals with familial disease. In that study the relationship of the affected relative to the index child did not affect seroconversion or progression rates [33].

As previously reported from the FPDR, children with familial type 1 diabetes carried the DR4-DQ8 risk haplotype more often than children with sporadic disease [4], especially those with an affected father. Such a difference was not seen in the prevalence of the DR3-DQ2 risk haplotype. On the contrary, the proportion of genotypes not including DR3-DQ2 and/or DR4-DQ8 was significantly reduced among children with familial disease, as described previously by Veijola et al [8]. In their data, the highest frequency of the DR3/DR4 genotype was seen in children with an affected sibling, while the DR4/x genotype was most common in children with an affected parent. Although these findings were non-significant, the current results are in line with those observations. Vadheim et al found that fathers with the DR4 allele transmit this allele more often to their offspring than DR4-positive mothers, suggesting that a preferential inheritance of the DR4 allele in children of affected fathers could explain the increased incidence of type 1 diabetes in the offspring of an affected father [14]. Similarly, high-risk HLA haplotypes were more likely to be transmitted to the offspring from a father than a mother with type 1 diabetes in a study by Tuomilehto-Wolf et al [15]. Although the findings potentially indicate that the affected father is characterised by an increased susceptibility for transferring certain high-risk haplotypes to their offspring, interpretations should be made with caution, since studies comparing the association between different affected family members and the HLA genotype in the index individual are sparse and the study populations in previous studies have usually been small.

When comparing index children with an affected parent diagnosed before vs after the birth of that child, we found no differences in the sex distribution of the offspring. In contrast, a higher male:female ratio among the affected parents was observed, but only if the parent was diagnosed before the birth of the index child. Similar results have been reported by Lorenzen et al, although their subpopulation was relatively small [29]. These findings support the hypothesis, first proposed by Warram et al [1], of a protective effect of maternal insulin treatment during pregnancy by inducing tolerogenic mechanisms to insulin. Exogenous insulin is transferred extensively to the fetus through the antigen–antibody complexes present in most insulin-treated mothers [34]. Similar differences have not been observed, however, in all studies [35]. In the BABYDIAB study, children with a father affected by type 1 diabetes developed positive autoantibodies earlier and at a higher frequency than children with an affected mother [36]. Thus, male sex of the affected parent seems to more strongly influence the initiation of autoimmunity to diabetes-associated autoantigens than disease progression. The longer history and experience of type 1 diabetes in affected parents diagnosed before the birth of the index child may explain the lower HbA_1c_ values in these index children at diagnosis, compared with those whose parents were diagnosed after their birth. However, similar differences were not seen in other metabolic values, probably because of limited statistical power.

The large study population of almost 5000 newly diagnosed children, derived from a nationwide register in the country with the highest incidence of type 1 diabetes, is a clear strength of our study. However, the retrospective design can be considered a limitation. Information on the presence of a family history of diabetes in relatives and the type of diabetes (type 1/type 2/gestational diabetes/other) was collected directly from the participating families using a questionnaire, which may possibly have led to some misclassifications. However, the risk of bias as to the family history of FDRs may have been smaller than if the extended family had also been included. Another source of selection bias is the inclusion criterion requiring sample availability for autoantibody and HLA analyses, although the frequency of children with familial and sporadic disease did not differ between those included and excluded [20].

In conclusion, the higher frequency of ketoacidosis and greater weight loss at diagnosis in the offspring of an affected father, in addition to fathers’ susceptibility to transferring the disease to their offspring more often than mothers, suggest that paternal type 1 diabetes seems to be associated with more severe disease in the offspring. This suggests that the children of fathers with type 1 diabetes might have a higher risk of diabetic complications than those of affected mothers. However, no studies to date have addressed that issue. No differences were observed in the autoantibody profile at diagnosis. The DR4-DQ8 haplotype was more frequent in children with familial compared with sporadic disease, and especially among those with a father affected by type 1 diabetes. Both genetic and environmental factors have been implicated to explain the higher incidence of type 1 diabetes in children with an affected father than in those with an affected mother. The sex difference seen between affected parents diagnosed before and after the birth of the index child supports the hypothesis that maternal type 1 diabetes protects against the development of type 1 diabetes in children.

## Electronic supplementary material

ESM (PDF 155 kb)

## Data Availability

The data generated and analysed during the current study are available from the corresponding author upon reasonable request.

## Abbreviations

DiMe: Childhood Diabetes in Finland
FDR: First-degree relative
FPDR: Finnish Pediatric Diabetes Register
GADA: Antibodies to GAD
IAA: Insulin autoantibodies
IA-2A: Antibodies to islet antigen 2
ICA: Islet cell antibodies
ZnT8A: Zinc transporter 8 autoantibodies
