# Supplementary material for: Characteristics of familial type 1 diabetes: effects of the relationship to the affected family member on phenotype and genotype at diagnosis
Source: Diabetologia. 2019 Jul 25;62(11):2025–39. doi: 10.1007/s00125-019-4952-8 (PMC6805821; doi:10.1007/s00125-019-4952-8)
Supplement: Supplementary file 1 — (PDF 155 kb) [file 125_2019_4952_MOESM1_ESM.pdf]

Electronic supplementary material

This appendix has been provided by the authors to give readers additional information about their work

Supplement to: Turtinen M, Härkönen T, Parkkola A, Ilonen J, Knip M, and Finnish Pediatric Diabetes Register. **Characteristics of familial type 1 diabetes: Effects of the relationship to the affected family member on the phenotype and genotype at diagnosis**

ESM Methods

Autoantibody assays

The cut off limits for antibody positivity were 2.80 relative units (RU) for IAA, 5.36 RU for GADA, 0.77 RU for IA-2A and 0.50 RU for ZnT8A based on the 99th percentiles in more than 350 Finnish nondiabetic children and adolescents. Between 2003 and 2016, the disease sensitivities and specificities of these assays were 42-62% and 92-99% for IAA, 64-90% and 90-98% for GADA, 62-72% and 93-100% for IA-2A and 48-70% and 97-100% for ZnT8A 6.2 in the Diabetes Autoantibody Standardization Programs and the Islet Autoantibody Standardization Programs. The detection limit for ICA was 2.5 Juvenile Diabetes Foundation units (JDFU).

HLA typing

The DQA1*05-DQB1*02 haplotype was shortened as DR3-DQ2 and the HLA-DRB1*04:01/2/4/5-DQB1*03:02 haplotype as DR4-DQ8. The HLA susceptibility for each study subject was classified based on comparison of genotype frequencies between 2991 children with type 1 diabetes and their affected family based artificial controls formed from haplotypes not transmitted to the diabetic child. The susceptibility was graded into six risk groups from strongly decreased to high (risk group 0-5).

ESM Results

Birth order and number of children in the family

In 1006 families (21.1%), the index child was the only child at diagnosis of type 1 diabetes. The index child was the first born more often if they had an affected mother compared with sporadic cases (ESM Table 1). No such a difference was seen between index cases with an affected father and those with an affected mother or between those with an affected father and the sporadic cases. The number of children in the family at the time of diagnosis of an index case was similar between these three groups.

HLA genotypes

Because of the higher proportion of boys with a father with type 1 diabetes in comparison to boys with an affected mother, we compared the prevalence of susceptible and protective and HLA genes in these two groups to assess the frequency of various risk genotypes in boys with an affected father and in boys with an affected mother. However, no differences were found between the two groups of boys (susceptible genotypes: 83.4% vs. 79.4, neutral and protective genotypes 16.6% vs. 20.6%; $p = 0.476$).

ESM Table 1. The number of children in the family at diagnosis of an index child and the birth order of the index case in relation to the presence of affected father or mother compared to sporadic cases.

	1. Affected father, n=253	2. Affected mother, n=141	3. Sporadic, n=4474	*p* value
Number children				0.106
1, %	20.9	27.7	20.4	
2, %	47.4	46.8	43.9	
3, %	22.1	19.9	23.2	
4, %	6.7	3.5	7.4	
5-14, %	2.8	2.1	5.1	
Birth order of the index child				0.023*
1st, %	54.2	56.7	48.5	
2nd, %	29.2	34.0	32.9	
≥ 3th, %	16.6	9.2	18.6	
				2 vs. 3: <0.05

Cross tabulation and χ^2 test was used for comparing frequencies.
p values: $^*p < 0.05$

The Finnish Pediatric Diabetes Register comprises the following investigators:

***Principal Investigator*:** Mikael Knip (Children's Hospital, Helsinki University Hospital, Helsinki, Finland)

Steering Committee: Per-Henrik Groop (Folkhälsan Research Center, Helsinki, Finland), Jorma Ilonen (Immunogenetics Laboratory, University of Turku, Turku, Finland), Timo Otonkoski (Children's Hospital, Helsinki University Hospital, Helsinki, Finland), Riitta Veijola (Department of Pediatrics, Oulu University Hospital, Oulu, Finland).

Locally responsible investigators:

Alar Abram (Department of Pediatrics, Kanta-Häme Central Hospital, Hämeenlinna, Finland), Henrikka Aito (Department of Pediatrics, HUS Porvoo Hospital, Porvoo, Finland), Ivan Arkhipov (Department of Pediatrics, Mehiläinen Länsi-Pohja Central Hospital, Kemi, Finland), Elina Blanco-Sequeiros (Department of Pediatrics, Central Ostrobothnia Central Hospital, Kokkola, Finland), Jonas Bondestam (Department of Pediatrics, HUS Lohja Hospital, Lohja, Finland), Markus Granholm (Department of Pediatrics, Jakobstad Hospital, Jakobstad, Finland), Maarit Haapalehto-Ikonen (Department of Pediatrics, Rauma Hospital, Rauma, Finland), Torsten Horn (Department of Pediatrics, Central Hospital of Central Finland, Jyväskylä, Finland), Hanna Huopio (Department of Pediatrics, Kuopio University Hospital, Kuopio, Finland), Joakim Janer (Department of Pediatrics, HUS Raasepori Hospital, Raasepori, Finland), Christian Johansson (Department of Pediatrics, Åland Central Hospital, Åland, Finland), Liisa Kalliokoski (Department of Pediatrics, Kainuu Central Hospital, Kajaani, Finland), Päivi Keskinen (Department of Pediatrics, Tampere University Hospital, Tampere, Finland), Anne Kinnala (Department of Pediatrics, Turku University Central Hospital, Turku, Finland), Maarit Korteniemi (Department of Pediatrics, Central Hospital of Lapland, Rovaniemi, Finland), Hanne Laakkonen (Department of Pediatrics, HUS Hyvinkää Hospital, Hyvinkää, Finland), Jyrki Lähde (Department of Pediatrics, Satakunta Central Hospital, Pori, Finland), Päivi Miettinen (HUS New Children's Hospital, Helsinki, Finland). Päivi Nykänen (Department of Pediatrics, Mikkeli Central Hospital, Mikkeli, Finland), Erik Popov (Department of Pediatrics, Vaasa Central Hospital, Vaasa, Finland), Mari Pulkkinen (Department of Pediatrics, HUS Jorvi Hospital, Espoo, Finland), Maria Salonen (Department of Pediatrics, Kymenlaakso Central Hospital, Kotka, Finland), Pia Salonen (Department of Pediatrics, Päijät-Häme Central Hospital, Lahti, Finland), Juhani Sankala (Department of Pediatrics, Savonlinna Central Hospital, Savonlinna, Finland), Virpi

Sidoroff (Department of Pediatrics, North Karelia Central Hospital, Joensuu, Finland), Anne-Maarit Suomi (Department of Pediatrics, South Ostrobothnia Central Hospital, Seinäjoki, Finland), Tuula Tiainen (Department of Pediatrics, South Karelia Central Hospital, Lappeenranta,Finland), Riitta Veijola (Department of Pediatrics, Oulu University Hospital, Oulu, Finland)
